# CD40/CD40L and Related Signaling Pathways in Cardiovascular Health and Disease—The Pros and Cons for Cardioprotection

**DOI:** 10.3390/ijms21228533

**Published:** 2020-11-12

**Authors:** Steffen Daub, Esther Lutgens, Thomas Münzel, Andreas Daiber

**Affiliations:** 1 Department of Cardiology, University Medical Center of the Johannes Gutenberg-University Mainz, 55131 Mainz, Germany; steffen.daub@unimedizin-mainz.de (S.D.); tmuenzel@uni-mainz.de (T.M.); 2 Experimental Vascular Biology Division, Department of Medical Biochemistry, University of Amsterdam, Amsterdam Cardiovascular Sciences, Amsterdam University Medical Centers, 1105 AZ Amsterdam, The Netherlands; e.lutgens@amsterdamumc.nl; 3 Institute for Cardiovascular Prevention (IPEK), Ludwig-Maximilians Universität, 80336 Munich, Germany; 4 German Center for Cardiovascular Research (DZHK), Partner Site Rhine-Main, Mainz, Germany and Partner Site Munich Heart Alliance, 80336 Munich, Germany; 5 German Center for Cardiovascular Research (DZHK), Partnersite Rhine-Main, 55131 Mainz, Germany

**Keywords:** CD40, CD40 ligand, atherosclerosis, inflammation, cardiovascular disease

## Abstract

The CD40–CD40 ligand (CD40L) dyad represents a scientific and clinical field that has raised many controversies in the past and cannot be clearly defined as being an either beneficial or harmful pathway. Being crucially involved in physiological immunological processes as well as pathological inflammatory reactions, the signaling pathway has been recognized as a key player in the development of both autoimmune and cardiovascular disease. Even though the possibilities of a therapeutic approach to the dyad were recognized decades ago, due to unfortunate events, detailed in this review, pharmacological treatment targeting the dyad, especially in patients suffering from atherosclerosis, is not available. Despite the recent advances in the treatment of classical cardiovascular risk factors, such as arterial hypertension and diabetes mellitus, the treatment of the associated low-grade inflammation that accounts for the progression of atherosclerosis is still challenging. Low-grade inflammation can be detected in a significant portion of patients that suffer from cardiovascular disease and it is therefore imperative to develop new therapeutic strategies in order to combat this driver of atherosclerosis. Of note, established cardiovascular drugs such as angiotensin-converting enzyme inhibitors or statins have proven beneficial cardiovascular effects that are also related to their pleiotropic immunomodulatory properties. In this review, we will discuss the setbacks encountered as well as new avenues discovered on the path to a different, inflammation-centered approach for the treatment of cardiovascular disease with the CD40–CD40L axis as a central therapeutic target.

## 1. Introduction

**Figure 1:**
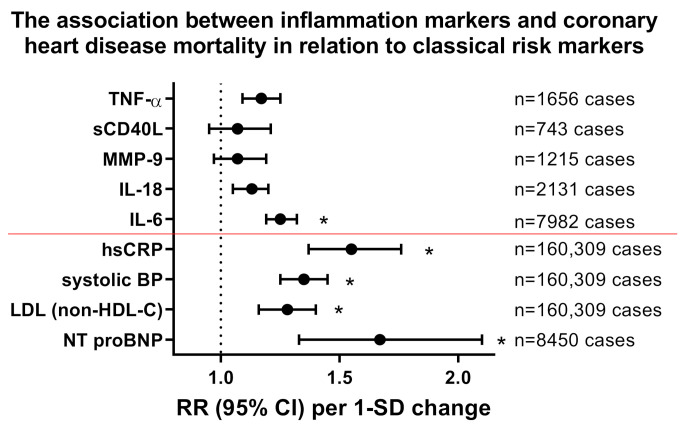
Impact of inflammation markers on cardiovascular mortality and comparison to classical/established cardiovascular risk markers. Relative risk (RR) for all coronary heart disease mortality in correlation with markers of inflammation interleukin (IL)-6, IL-18, matrix metalloproteinase (MMP)-9, soluble CD40 ligand (sCD40L or CD154) and tumor necrosis factor (TNF)-α obtained by meta-analysis with adjustment for age, sex, smoking status, adiposity markers, blood pressure and/or lipid markers (number of cases as indicated). Inflammatory risk markers were compared to classical cardiovascular risk markers such as hsCRP, systolic blood pressure (BP), low density lipoprotein (LDL, measured as non-HDL-C) and N-terminal fragment of natriuretic peptide type B (NT proBNP). The classical risk markers are all below the red line. Risk increases are shown per 1-SD changes of cytokines and classical cardiovascular risk markers. * indicates significant differences to control group. Redrawn from tabular data in [14] for inflammation markers, [17,18] for hsCRP/systolic BP/LDL (measured as non-HDL-C) and [19] for NT proBNP.

Cardiovascular disease (CVD) and its main driver, atherosclerosis, have been recognized as inflammatory-driven entities [1,2]. Starting with the initiation of an atherosclerotic plaque by macrophage-releasing cytokines, which in turn prompt vascular endothelial cells to attract leucocytes that drive the maturation and destabilization of the atherosclerotic lesion [3], low-grade inflammation fuels every step on the path to clinical manifestations of cardiovascular disease such as myocardial infarction or stroke. Within the last 50 years, clinical efforts have focused on treating risk factors associated with CVD such as smoking, lifestyle changes, hypertension and dyslipidemia. This approach, including the use of lipid-lowering drugs, such as 3-hydroxy-3-methylglutaryl coenzyme A (HMG-CoA) reductase-inhibitors (statins) and proprotein convertase subtilisin-kexin type 9 (PCSK9) inhibitors, has reduced the risk of CVD by 30% [4]. Despite this therapeutic success, the majority of the population still suffers from atherosclerotic complications while showing signs of systemic low-grade inflammation [5] indicated by elevated plasma levels of high-sensitive C-reactive protein (hsCRP) [6]. The PROVE IT-TIMI consortium showed that high CRP levels are indicative of myocardial infarction-induced death in patients with acute coronary syndrome [7]. Recent data from the Genome-Wide Association Study (GWAS) identified several risk loci that are linked to cardiovascular inflammation, thereby supporting that inflammation represents a major cardiovascular risk factor [8,9]. Chronic inflammatory diseases (e.g., systemic lupus erythematosus, rheumatoid arthritis and psoriasis) show an association with higher cardiovascular risk [10,11,12,13]. In line with this, a number of circulating cytokines and soluble CD40 ligand (sCD40L) were established as independent cardiovascular risk factors (Figure 1) [14,15] and therapeutic targeting of these cytokines showed beneficial effects in various studies and models [16]. This is corroborated by more traditional biomarkers for the progression of cardiovascular disease and death attributable to vascular dysfunction, namely hsCRP, systolic blood pressure (SBP) and non-HDL lipoproteins (in lieu of low-density lipoproteins) as recently reviewed in a meta-analysis comprising approximately 160,000 patients and a staggering 1.3 million person-years of follow-up [17,18], as well as N-terminal fragment of natriuretic peptide type B (NT proBNP) as recently reviewed in a meta-analysis comprising approximately 8400 patients (Figure 1) [19]. However, one should note that the classical cardiovascular risk markers obviously have a more powerful predictive value in comparison with inflammatory markers and accordingly may provide better risk assessment in the clinical situation.

## 2. Targeting Inflammation in Patients Decreases Cardiovascular Risk

Corroborating this, in 2017, the Canakinumab Anti-inflammatory Thrombosis Outcomes Study (CANTOS) employed canakinumab, a drug designed to bind and neutralize interleukin 1β, which improved cardiovascular outcome in patients with a history of CVD and elevated hsCRP-levels [16,20]. Despite showing a clear correlation between treatment-induced hsCRP reduction and improved cardiovascular outcome, this approach was deemed too cost-ineffective and has since not been revisited. Furthermore, in the setting of diabetes, canakinumab therapy showed an improvement of cardiovascular prognosis [21]. Studies with other IL-1 receptor blockers or antagonists such as Anakinra, rilonacept and gevokizumab are underway to investigate their beneficial effects in patients with cardiovascular disease [22]. In addition, the COLCOT study showed that unspecific anti-inflammatory therapy by colchicine may have cardioprotective effects [23], which was later corroborated by the LoDoCo2 trial which found a reduction in cardiovascular events in patients suffering from CVD who received a daily, low dose of colchicine [24]. It is also well established that cardiovascular prognosis can be improved by targeted anti-inflammatory therapy in patients with rheumatoid arthritis (IL-6, TNF-α and IL-17A cascades) [25,26], psoriasis (IL-17/IL-23 axis) [27,28,29] and systemic lupus erythematosus (IL-17A signaling) [30] and other markers of inflammation, even beyond the inflammasome [24,31].

Importantly, established cardiovascular drugs have known pleiotropic anti-inflammatory effects that improve cardiovascular prognosis as shown for angiotensin converting enzyme (ACE) inhibitors [32,33], angiotensin-II receptor type 1 blockers [34,35] and statins [7,36,37]. Furthermore, the vital interaction/crosstalk between inflammation, thrombosis and coagulation contributes to higher cardiovascular risk [38] and thrombosis/coagulation are therefore pharmacologically targeted, e.g., by rivaroxaban/aspirin therapy [39] or FXI antagonization [40,41]. This is highly important as CD40–CD40L signaling also represents a key mechanism of platelet activation and downstream thrombotic and coagulant pathways.

## 3. Mechanistic Background of CD40–CD40L Signaling

As a key player in immunity, the CD40–CD40L dyad regulates T cell activation, cytokine production and isotype switching [42]. CD40, a member of the tumor necrosis factor (TNF) receptor superfamily, was discovered in 1984 on B lymphocytes [43]. The 54kDa protein, expressed on immune cells, such as B cells, dendritic cells and macrophages, as well as non-immune cells, such as endothelial cells, vascular smooth muscle cells and fibroblasts [42], acts as a receptor and upon activation by its classical ligand, CD40 ligand (CD40L, CD154, 39kDa protein), trimerization and receptor internalization are induced. In patients who suffer from an interruption in CD40–CD40L interaction within the Hyper-IgM syndrome, lethal infections due to impaired T- and B-cell-driven immune response are common [44,45]. The CD40L–CD40 dyad is also involved in the pathogenesis of numerous inflammatory and autoimmune diseases such as systemic lupus erythematosus, diabetes type 1, rheumatoid arthritis and allograft rejection [46]. CD40L is a member of the TNF receptor superfamily and is expressed on T cells and platelets upon their activation [47,48]. Of note, almost 90% of circulating sCD40L is released from activated platelets [49]. Accordingly, the usefulness of sCD40L as a marker of vascular inflammation is subject to vital scientific discussions.

**Figure 2:**
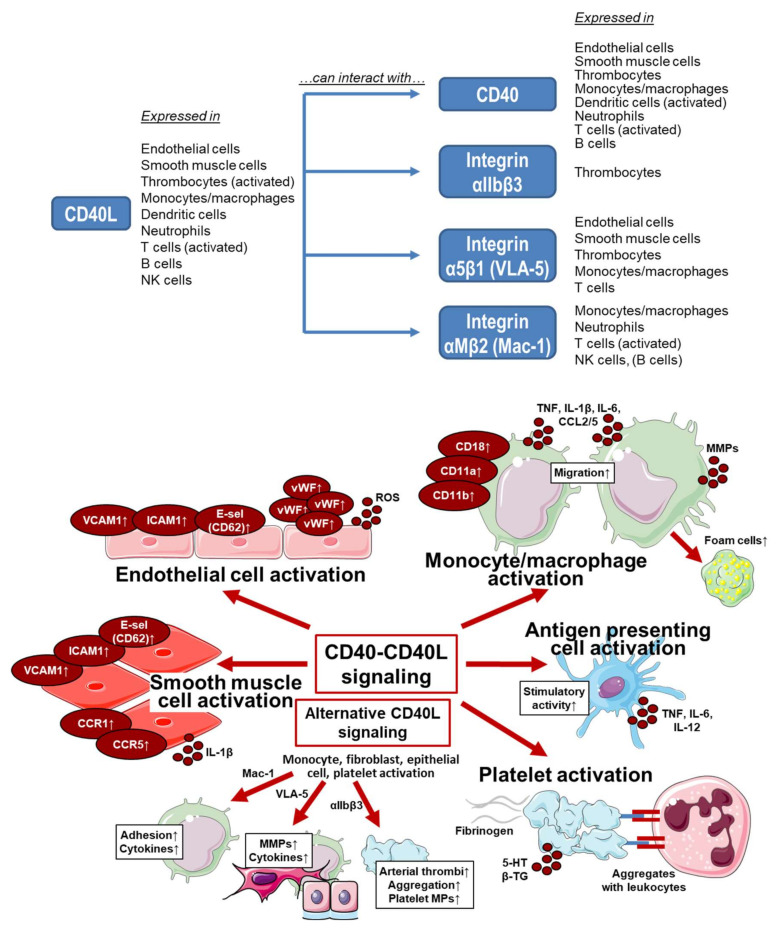
**Upper panel:** Overview on the expression of CD40L and its molecular targets and receptors in different cell types as well as potential interactions. Summarized from information in [52,61,62,63,64]. **Lower panel:** Effects on CD40–CD40L and alternative signaling on different cell types. VCAM, vascular cell adhesion molecule; ICAM, intercellular adhesion molecule; E-sel, E-selectin (CD62); vWF, von Willebrand factor; ROS, reactive oxygen species; CD, cluster of differentiation; CD11a, integrin αL; CD11b, integrin αM; CD18, integrin Mac-1; TNF, tumor necrosis factor; IL, interleukin; CCL, CC-chemokine ligand; MMPs, matrix metalloproteinases; CCR, CC chemokine receptor; VLA-5, fibronectin receptor; αIIbβ3, glycoprotein GPIIb/IIIa (CD41/CD61), an integrin; MPs, microparticles; 5-HT, serotonin; β-TG, β-thromboglobulin. Summarized from table in [61] with permission. Contains images from Servier Medical Art by Servier, licensed under a Creative Commons Attribution 3.0 Unported License.

Besides this key role for inflammatory processes, CD40–CD40L signaling plays a key role for thrombosis [42,50] and contributes to the coagulation process (e.g., via tissue factor production) [51], where also the activation of the endothelium and smooth muscle cells plays an important role [52]. Whereas the CD40L-mediated activation of immune cells likely proceeds via its molecular targets and receptors CD40 and the integrin complexes *α*_5_*β*_1_ and Mac-1 [53,54,55,56,57], the CD40L-mediated activation of thrombocytes proceeds by its binding to CD40 [57] as well as the thrombocyte-specific integrin receptor *α_IIb_β*_3_ [58,59]. For expression of CD40L and its molecular targets and receptors in different cell types as well as the cellular effects of these signaling pathways see Figure 2. Extensive research has been conducted regarding CD40(L)-directed immunotherapy in cancer, recently reviewed by Li and colleagues [60]. In the treatment of cancer, strategies involving the CD40–CD40L dyad aim to elicit immune cell activation directed against the tumor, resulting in an antitumor immune effect [60].

It is important to note that CD40 as a receptor has no intrinsic capabilities of signal transduction. This takes place via the recruitment of tumor necrosis factor receptor-associated factors (TRAFs) for which the receptor contains distinct cytoplasmic binding sites: a proximal one for TRAF6 and a distal one for TRAF2/3/5 [65]. Depending on the TRAFs recruited, CD40 activates downstream pathways such as NF-κB, C-Jun N-terminal kinase (JNK) or the p38 mitogen-activated protein (MAP) kinase pathway [66]. Apart from its classical binding partner, CD40 is also activated by C4b-binding protein (C4BP), which is physiologically present within the germinal centers of secondary lymphoid follicles where it interacts with CD40 on B cells in order to induce proliferation [67]. Lastly, heat-shock protein (HSP) 70 has been shown to bind and activate CD40 on myeloid cells [68].

Notably, CD40L also appears in a soluble form (sCD40L), cleaved from its membrane-bound counterpart. After secretion by activated platelets and T cells, the soluble molecule retains the capability to bind and activate the aforementioned receptors [69]. Soluble CD40L has been investigated as a potential marker for future cardiovascular events which has, to this day, yielded very mixed results. As this was attributed to multiple factors, including the possible circadian rhythmicity and stability of sCD40L in combination with different anticoagulants that are used in the clinical setting, the predictive value of sCD40L remains unclear [70]. However, in several studies, sCD40L is a strong predictor of cardiovascular events, indicating that sCD40L is a relevant biomarker in several subgroups of patients. Further extensive studies are needed to identify the characteristics of these subgroups with high sCD40L levels (i.e., diabetes mellitus and hypertension or maybe high hsCRP levels). These patients with a high burden of sCD40L might then profit from CD40L blockade as described in this review.

## 4. CD40–CD40L Dyad in Cardiovascular Diseases

### 4.1. Dyad in Atherosclerosis, Evidence from Experimental Models

Through the course of the development of atherosclerosis, the CD40–CD40L dyad is involved in multiple steps that ultimately lead to plaque destabilization and rupture (Table 1). Primary initiation of the atherosclerotic plaque is brought on by platelet-bound CD40L that promotes the formation of ultra-large von Willebrand factor (ULVWF) multimers as shown by Popa and colleagues in human umbilical vein endothelial cells (HUVECs) [71]. These highly thrombogenic molecules then facilitate platelet tethering, monocyte recruitment and extravasation as initial steps in the formation of atherosclerotic lesions [71]. Another CD40L-dependent mechanism that greatly contributes to the formation of atherosclerotic plaques is the ligand’s ability to stimulate CD40-bearing endothelial cells. In an experimental model employing HUVECS, CD40L (in combination with interleukin-4) greatly increased the expression of VCAM-1 and P-selectin, which, in turn, facilitates the adhesion of leukocytes [72]. The third mechanism by which the CD40–CD40L dyad contributes to initial plaque formation was recently described by Gerdes and colleagues [70]. In their animal model utilizing CD40^−/−^ApoE^−/−^ mice, the formation of platelet-leukocyte aggregates was shown to depend on platelet CD40 signaling. Taken together, these three CD40(L)-dependent mechanisms crucially promote leukocyte recruitment and vascular transmigration thereby establishing the early stages of the atherosclerotic plaque. On a mechanistic basis, CD40(L) drives the differentiation of monocytes into macrophages and foam cells [73] and the production of inflammatory molecules, before eliciting the production of matrix metalloproteinases (MMP) which ultimately cause plaque rupture [65].

The TRAFs known to elicit CD40 signaling appear to show fundamental differences regarding their involvement in atherosclerosis and metabolic disorders. CD40–TRAF6 interaction was shown to be crucial to neointima formation in mice carrying a targeted mutation that prevented CD40–TRAF6 signaling. No beneficial effect was observed in response to a defect in CD40–TRAF2/3/5 binding [74]. Subsequently, inhibition of TRAF6, but not TRAF2/3/5, resulted in an anti-inflammatory phenotype and a reduction in atherosclerotic lesions in apolipoprotein E deficient (ApoE^−/−^) mice [75]. Finally, TRAF6 inhibition, in contrast to the inhibition of TRAF2/3/5, elicited protective effects in an experimental model for obesity-induced insulin resistance as described below. Taken together, these findings suggest a crucial role for the CD40–TRAF6 axis in the development of atherosclerosis mainly via macrophage-driven inflammation, while the CD40–TRAF2/3/5 axis seemingly has a more balanced role in the modulation of immunological processes (e.g., isotype switching and activation of B cells) [76]

### 4.2. Dyad in Myocardial Infarction, Heart Failure

As atherosclerosis is also present in the coronary arteries, and plaque rupture can lead to myocardial infarction, the CD40/CD40L dyad is intimately involved in the pathophysiology of the underlying disease (Table 1). Indeed, patients suffering from myocardial infarction also have elevated sCD40L levels and two studies demonstrated a correlation between sCD40L serum levels and detrimental cardiovascular outcomes [80,81]. Additionally, sCD40L levels were found to be elevated in patients suffering from acute or chronic heart failure [82]. In their study, the authors found elevated serum levels of sCD40L as well as differing levels in the blood compartments analyzed. It was found that the highest concentration of the soluble ligand was present in the coronary sinus of the patients which suggests that the heart is capable of producing sCD40L [82]. The effects of a CD40 blockade on cardiac remodeling in pressure overload induced heart failure is detailed in the following section.

## 5. Traditional Approaches

### 5.1. General CD40(L) Blockade

#### 5.1.1. CD40^−/−^ Animals and a General CD40 Blockade

A general blockade of CD40 comes with the risk of severe immunosuppression as is evident in patients suffering from the Hyper-IgM syndrome described above [44]. Mice that completely lack CD40 are indeed known to exhibit impaired immunoglobulin class-switching as they lack a proper IgG/IgA-driven immune response as well as the formation of germinal centers [83]. Bleeding complications were also observed in CD40(L) knockout mice [84]. Pharmacological blockade of the CD40 receptor by antagonizing antibodies has for the most part been studied in animal models that mimic autoimmune disease. A recent study observed beneficial effects in a mouse model for systemic lupus erythematosus in which CD40 blockade by an antagonizing antibody reduced proteinuria in lupus nephritis [85].

#### 5.1.2. CD40L^−/−^ Animals and a General CD40L Blockade

In a model for arterial hypertension utilizing a steady infusion of angiotensin II, CD40L-deficiency greatly blunts the development of endothelial dysfunction and reverses the prothrombotic state that is observed in wild-type animals [77]. CD40L-deficiency also prolonged bleeding time and decreased the leukocyte-platelet-aggregates that were increased by angiotensin II infusion. In another study, our group could show the protective effects of CD40L-deficiency in animals subjected to a high-fat diet. In this obesity and type 2 diabetes mellitus model, CD40L^−/−^ mice were protected from obesity-associated vascular inflammation, oxidative stress and endothelial dysfunction [78]. Despite these beneficial effects, blocking CD40L must be regarded as a double-edged sword: The interaction between CD40L and αIIbβ3 was shown to be crucial for the process of thrombus stabilization [58]. André and colleagues assessed the stability of thrombi in mesenteric arterioles of mice and found that the thrombi that developed in CD40L^−/−^ mice were prone to rupture and embolization. While hemostasis was not impaired in these animals, CD40L-αIIbβ3 interaction was found to be essential to platelet function and thus thrombus stabilization [58]. In accordance with these findings, Kawai and colleagues found multiple thromboses in their allograft model [86]. In non-human primates who were administered an antibody raised against CD40L, while this improved renal allograft survival, four out of nine animals showed thromboembolic complications (two renal arterial thromboses, one renal vein thrombosis and one mesenteric artery thrombosis). While these side effects were shown to be prevented by the administration of heparin, together with the aforementioned study by André and colleagues, these findings would lead to a discontinuation of clinical trials investigating CD40L blockade in humans and prevented further studies for years, as detailed in Section 3 of this review.

### 5.2. Novel Approaches Targeting the CD40–CD40L Dyad

#### 5.2.1. TRAF-STOPS

Recently, in order to circumvent side effects of general CD40(L) blockade, small molecules that are capable of inhibiting the interaction between CD40 and TRAF6, termed TRAF-STOPs, have been developed. In 2014, a small molecule CD40-TRAF6 inhibitor (6877002) was selected using an approach for virtual ligand screening (VLS) based on in silico structures. Obtaining the X-ray structures of both the CD40 and TRAF6 molecules, a small molecule was chosen by combining in silico and in vitro selection methods (described in great detail in [87]). Afterwards, the 6877002 compound was subjected to in vivo testing in disease-specific animal models. Chatzigeorgiou and colleagues described 6877002′s effects in an established animal model for obesity-induced insulin resistance (diet-induced obesity, DIO) [88]. After inducing obesity in C57BL/6 by feeding them a high-fat diet (HFD, 20% kcal carbohydrate, 60% kcal fat, 20% kcal protein) for six consecutive weeks, the animals were treated with the CD40-TRAF6 inhibitor for another six weeks while receiving HFD. While leaving the expected gain in body weight similar between the groups, 6877002 improved insulin sensitivity compared to mice treated with vehicle [88]. Furthermore, DIO-induced invasion of CD11b+F4/80+CD11c+ (M1) macrophages into the adipose tissue as well as the development of hepatosteatosis were blunted. In summary, the small molecule inhibitor demonstrated beneficial effects on adipose tissue inflammation and insulin resistance in this therapeutic setting after the induction of obesity by a high-fat diet.

Another animal model in which TRAF-STOPs have been successfully tested is pressure overload induced heart failure [89]. In 2019, Bosch and colleagues showed that treatment with TRAF-STOPs reduced left ventricular mass and cardiac hypertrophy in C57BL/6 mice six weeks after transverse aortic constriction. Histologically, the hypertrophied myocardium showed a reduction in inflammatory cells (lymphocytes and macrophages) as well as ameliorated cardiac fibrosis.

The small molecule CD40-TRAF6 interaction inhibitor 6877002 was subsequently tested in atherosclerotic ApoE^−/−^ mice. Seijkens et al. were able to show that TRAF-STOPs were effective at reducing the atherosclerotic plaque formation in the aortic arch, the typical location of atherosclerosis in this particular animal model [90]. Furthermore, the atherosclerotic plaques of young ApoE^−/−^ mice treated with a CD40-TRAF6 inhibitor showed a reduction in fibrous cap atheromata and a reduction in infiltrating immune cells, namely Mac3^+^ macrophages, CD3^+^ T cells and Lyc6G^+^ neutrophils. The authors subsequently studied the effects of TRAF-STOPs on older ApoE^−/−^ mice that had already developed severe atherosclerosis. In these animals, the administered TRAF-STOPs halted atherosclerotic progression as evidenced by a reduction in inflammatory cells within the atherosclerotic plaque, which again contained fewer Lyc6G^+^ neutrophils, Mac3^+^ macrophages and CD3^+^ T cells [90]. Additionally, within the atherosclerotic plaques fewer necrotic cores were found and they exhibited an increase in collagen and αSMA^+^ smooth muscle cells, which evidences the transition from an active atherosclerotic lesion to a stable plaque elicited by TRAF-STOP treatment. Leukocyte recruitment was reduced in animals treated with a small molecule CD40-TRAF6 interaction inhibitor, resulting in dampened leukocyte rolling at the carotid arterial wall visualized by intravital microscopy. Of note, the two TRAF-STOPs used in this study did not alter hematologic parameters or overall leukocyte content in the blood of the treated animals, suggesting a lack of immunosuppressive side effects. This was corroborated by an adequate germinal center formation and antigen-specific immunoglobulin response in those animals.

Prompted by the marked reduction in macrophage activation within the atherosclerotic lesions caused by TRAF-STOPs, Seijkens and colleagues were able to utilize recombinant high-density lipoprotein nanoparticles which contained the aforementioned TRAF-STOP 6877002 (TRAFi-HDL) for macrophage-specific targeting [90]. These molecules were constructed from human apolipoprotein A-I and the phospholipids 1-myristoyl-2-hydroxy-sn-glycero-phosphocholine (MHPC) and 1,2-dimyristoyl-sn-glycero-3-phosphatidylcholine (DMPC) [87]. In the animals intravenously treated with nanoparticles, the effects of a direct TRAF-STOP administration were for the most part reproduced, while dosage and application frequency could be reduced. In a translational study, these TRAF-STOP-incorporating HDL nanoparticles (TRAFi-HDL) were safely tested in non-human primates [91]. Lameijer and colleagues were able to show the previously described accumulation of the nanoparticle in the atherosclerotic lesions within the aortic sinus in cynomolgus monkeys (Macaca fascicularis) as well as mice. Zirconium-89-radiolabeled TRAFi-HDL was found to accumulate primarily in the liver, spleen and kidneys shortly after injection with the largest amount of injected dose that was found to be traceable in the liver, spleen and the kidneys after sacrificing the non-human primates after 72 h. In mice and non-human primates, TRAFi-HDL did not alter the count of erythrocytes or lymphocytes in the blood, numbers of T and B cells in bone marrow and spleen, nor did it impact hepatic and kidney function. The only slightly elevated parameter reported in this regard was alkaline phosphatase. Lipids, glucose, protein and electrolytes were unaffected. TRAFi-HDL was found to leave a physiologic inflammatory response intact as serum cytokine and chemokine (Interleukin 6 and 1β, chemokine (C-C motif) ligand 2, tissue necrosis factor α and serum amyloid P-component) levels were unaltered.

#### 5.2.2. Silencing RNA

Micro-RNAs have been recognized as important regulators in many pathophysiological processes and are linked to cardiovascular disease and the progression of atherosclerosis [92,93]. Consequently, silencing RNA (siRNA)-mediated effects of CD40-inhibition were investigated by Hueso et al. in ApoE-deficient mice [94,95]. In these animals, a reduction in atherosclerotic lesion burden was elicited by the treatment with siRNA targeting CD40 along the whole aorta with emphasis on the aortic sinus. Again, inhibition of CD40 markedly reduced macrophage accumulation in the atherosclerotic lesions. The authors also found a reduction in NF-κB-positive cells, which have previously been reported to correlate with the severity of atherosclerotic lesions [96] in the intima of the mice treated with siRNA. As the method of CD40 signaling disruption in the form of siRNAs is a systemic approach, the authors found a reduction of CD3^+^CD40^+^ T lymphocytes as well as CD11b^+^CD40^+^ monocytes in the spleens of the animals treated with the silencing RNA, evidencing a systemic anti-inflammatory effect. While clearly demonstrating beneficial effects regarding the stability and progression of atherosclerotic lesions, in the same animals, however, siRNAs directed at CD40 created an inflammatory milieu in the kidneys [97]. In clear contrast to atherosclerotic lesions, silencing CD40 systemically increased macrophage infiltration in the kidney and led to a rise in content of NF-κB-positive cells. Accompanying these histological changes, capillary density was increased in kidneys of animals treated with siRNA. Lastly, and most devastatingly, the authors reported a marked increase in serum creatinine as a clinical readout for acute kidney injury as a side effect of siRNA therapy.

#### 5.2.3. Antisense Oligonucleotides

Antisense oligonucleotides (ASOs) targeting CD40 were first described by Gao et al. in 2005 [98] before being revisited by Arranz and colleagues in an animal model for colitis [99]. While Gao and colleagues were able to show anti-inflammatory properties of the first generation of ASOs, the compound used was applied as an enema reaching very high local concentrations within the inflamed intestines. As ASOs are by nature bulky and polar molecules that crucially depend on a suitable delivery vehicle, Arranz et al. used an ASO incorporated in amphoteric liposomes, nov038/CD40, which was intravenously injected into Balb/c mice that had been subjected to the induction of colitis by rectal administration of 2,4,6-Trinitrobenzenesulfonic acid (TNBS) [99]. In the animals, high blood concentrations of the ASO could be achieved, which prevented the weight loss normally observed after the induction of colitis in this model. A single treatment with nov038/CD40 was shown to eliminate all signs of histological damage, which typically manifests as thickening of the colon wall, ulcerations and fibrosis in these animals. Prednisolone, in comparison, was not able to revert the microscopic damage caused by the experimental colitis. In the serum, treatment with nov038/CD40 reduced levels of interleukin 6 and chemokine IP10. Next, Arranz and colleagues investigated mesenteric lymph nodes and spleens from animals treated with the ASO and found a reduction in CD11b^+^ macrophages as well as a suppression of the expansion of CD4^+^ and CD8^+^ t cells associated with the onset of experimental colitis. Leaving regulatory T cells as well as B cells unaffected, the authors concluded that the ASO, in contrast to glucocorticoids, did not induce general immunosuppression even when administered systemically. This was further corroborated by the generation of sufficient IgG antibody titers when animals that underwent treatment with nov038/CD40 were challenged with antigen ovalbumin. Lastly, Arranz et al. were able to show that ASO-treatment directed at CD40 also had a curative effect on experimental colitis that had already been established in mice for three days prior to the administration of nov038/CD40. In 2015, ASOs targeting CD40 were revisited in an experimental model for acute kidney injury [100]. In animals administered doxorubicin, ASOs were able to greatly reduce inflammation within the kidney, thereby ameliorating renal damage.

#### 5.2.4. Specific Blockade of the CD40L-Mac-1 Interaction

The non-classical interaction between CD40L and Mac-1 on monocytes has received particular attention due to its involvement in the formation of the atherosclerotic plaque. While a general blockade of Mac-1 is deemed too dangerous in the clinical setting as the molecule interacts with a multitude of immunological targets (e.g., ICAM-1 and RAGE), and genetic mutations that impair Mac-1 signaling cause immune defects (leucocyte adhesion deficiency), the specific blockade of CD40L-Mac-1 interaction with the antibody anti-M7 was able to successfully dampen inflammation in an acute model for peritonitis without causing immunosuppression [101]. By blocking the CD40L binding domain within the receptor molecule Mac-1 (EQLKKSKTL motif), Wolf et al. were able to show a reduction in leukocyte recruitment within inflamed mesenteric venules in wild-type mice, an effect that was diminished in Mac-1 knockout mice [102]. Furthermore, strategically blocking CD40L-Mac-1 interaction via Anti-M7 in an in vivo model of sterile sepsis, induced by lipopolysaccharides (LPS), reduced myeloid cell recruitment in the ensuing sepsis when administered within 2 h [102]. In summary, specific inhibition of CD40L-Mac-1 interaction succeeded in limiting myeloid cell recruitment to the atherosclerotic plaque while leaving host immunity intact [102].

## 6. Clinical Trials

Due to the dyad’s intimate involvement in (auto-)immunological physiological and pathological processes, clinical studies have so far focused on the treatment of inflammatory diseases. It is, however, prudent to closely observe the side effects in the wake of a general CD40(L) blockade.

### 6.1. Blocking CD40L

In patients suffering from proliferative lupus glomerulonephritis, BG9588, a humanized anti-human CD40L antibody, while showing beneficial immunomodulatory action, caused severe thromboembolic events (i.e., two myocardial infarctions) [103]. Assuming the drug had actually led to the destabilization of already present atherosclerotic lesions, the study was immediately halted. Investigations into the exact mechanism behind this severe side effect revealed that the thromboembolic events might have been caused by immune complexes comprising soluble CD40L and antiCD40L antibodies which in turn were shown to be able to activate platelets through Fc-receptor binding [104]. It was 12 years after the initial study, in 2015, that Shock and colleagues were able to synthesize an anti-CD40L antibody (CDP7657) that lacked the Fc domain, which was subsequently tested in healthy volunteers and patients with SLE [105,106,107]. As no thromboembolic events have so far been reported, the drug, now termed dapirolizumab pegol, is currently undergoing evaluation in a Phase III trial involving an estimated 450 participants (ClinicalTrials NCT04294667). IDEC-131, another antagonistic CD40L antibody, failed to demonstrate superiority over placebo in a Phase II trial [108]. In this study, no thromboembolic events were reported.

### 6.2. Blocking CD40

As a general blockade of CD40L-signaling has, as detailed above, resulted in severe thromboembolic events in both experimental models and human trials, an antagonistic CD40 blockade has been the main focus in patients suffering from inflammatory diseases and transplant rejection. Bleselumab (ASKP1240) was shown to be effective in the treatment of psoriasis [109] while demonstrating good tolerability without producing severe side effects. The drug is currently being evaluated in a Phase II trial (ClinicalTrials NCT02921789) as part of a therapy-regimen in glomerulosclerosis after kidney transplantation. BI 655064, another antagonistic anti-CD40 antibody was safely tested on healthy volunteers [110], yet failed to meet the primary endpoint (clinical improvement) in patients with rheumatoid arthritis [111]. It was, however, shown that the drug reduced levels of activated memory B cells (CD19+IgD-CD27+CD95+), inflammatory (IgG and A rheumatoid factor) and bone resorption biomarkers (RANKL). With its beneficial safety profile and no reported thromboembolic or cardiovascular events, BI 655064 is being evaluated in a Phase II trial in patients with lupus nephritis (ClinicalTrials NCT02770170). While the CD40 antibody ch5D12 was safely used in patients suffering from Crohn’s disease [112], demonstrating the ability to induce clinical remission, the agent has not been investigated in a wider clinical approach.

## 7. Outlook

As detailed in this review, while clinical trials investigating the effects of a general CD40 blockade have been undertaken, none of them have focused on cardiovascular outcome. With all trials involving patients suffering from autoimmune diseases, it seems the CD40–CD40L dyad has been forgotten by cardiologists. In the coming years, we will have the unique opportunity to see the effects of antagonizing CD40 with regards to underlying diseases as well as cardiovascular side-effects. As described, this systemic and broad approach might on the one hand prevent cardiovascular events, while on the other severely compromise physiological immune defense. Revisiting CD40L antagonistic antibodies, dapirolizumab pegol offers the chance to alleviate our (well-founded) fears regarding unwanted therapeutic plaque destabilization.

**Figure 3:**
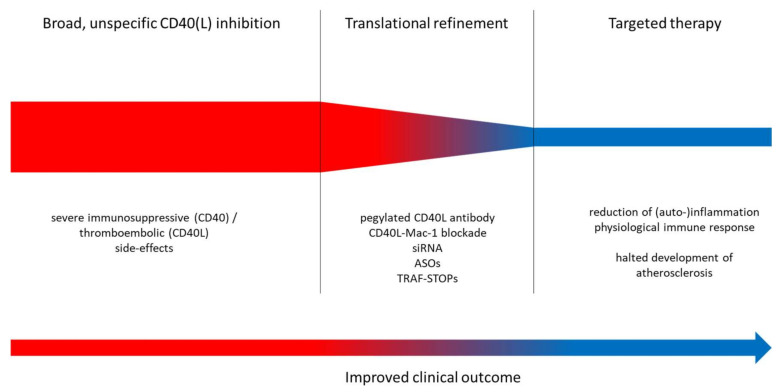

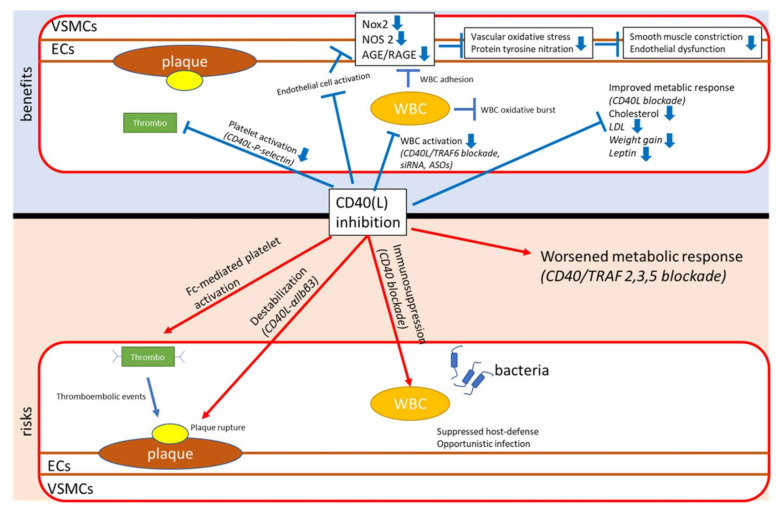
**Upper panel:** Proposed translational strategy for improved anti-inflammatory therapy using CD40–CD40L-antagonizing approaches. **Lower panel:** Key mechanisms of inflammation, atherothrombosis, oxidative stress and endothelial dysfunction. Improvement of these adverse key mechanisms by CD40(L)/TRAF inhibition. siRNA, small interfering RNA; ASOs, antisense oligonucleotides; TRAF-STOPs, inhibitors of TNF receptor associated factor; ECs, endothelial cells; VSMCs, vascular smooth muscle cells; NOX2, phagocytic NADPH oxidase (gp91phox); NOS2, inducible nitric oxide synthase; AGE, advanced glycation end products; RAGE, receptor for AGE; LDL, low density lipoprotein; WBC, white blood cell.

Despite all efforts, there is a possibility that a general, systemic CD40(L) blockade, while being suitable for the short-term treatment of exacerbations in autoimmune diseases, has limiting side effects in the long-term treatment of patients with cardiovascular disease (Figure 3). Using novel, groundbreaking approaches, therapy targeted at small, yet crucial aspects within the CD40–CD40L dyad, a translational strategy may ultimately end up creating a feasible and safe way to reduce the risk for cardiovascular events. In light of the powerful anti-inflammatory effects of antagonization of CD40–CD40L signaling (example provided for type 2 diabetes mellitus and obesity in Figure 3), it would be a waste of resources not to push forward the pharmacological exploitation of this important pathway.

## Figures and Tables

**Table 1:** Impact of CD40L–CD40 signaling on cardiovascular disease beyond atherosclerosis.

Species.	CVD Entity	Effect	Pathway	Ref
mouse	Atherosclerosis	Plaque initiation	CD40L-ULVWF	[71]
		Leucocyte recruitment/infiltration	CD40L-CD40 (ECs), VCAM-1	[72]
		Plaque destabilization/rupture	CD40L-Mac1	[73]
	Myocardial infarction	Thrombus stabilization/platelet activation	CD40L- αIIbβ3 Cd40L-CD40	[58]
	Arterial hypertension	Plaque formation	CD40L- dependent	[77]
	High-fat diet	Vascular inflammation	CD40L- dependent	[78]
human	Coronary artery disease	CD40 expression on endothelial cells, increased susceptibility to atherosclerosis	SNP rs1883832	[79]
	Acute coronary syndrome	Increased risk for death and recurrent MI	Elevated sCD40L levels in serum	[80]
	Myocardial infarction	Increased mortality	Elevated sCD40L levels in serum	[81]
	Heart failure	LV dysfunction	CD40L-CD40	[82]
